# Pituitary Adenomas: From Diagnosis to Therapeutics

**DOI:** 10.3390/biomedicines9050494

**Published:** 2021-04-30

**Authors:** Samridhi Banskota, David C. Adamson

**Affiliations:** 1 School of Medicine, Emory University, Atlanta, GA 30322, USA; samridhi.banskota@emory.edu; 2 Department of Neurosurgery, Emory University, Atlanta, GA 30322, USA; 3 Neurosurgery, Atlanta VA Healthcare System, Decatur, GA 30322, USA

**Keywords:** pituitary adenoma, prolactinoma, acromegaly, Cushing’s, transsphenoidal, CNS tumor

## Abstract

Pituitary adenomas are tumors that arise in the anterior pituitary gland. They are the third most common cause of central nervous system (CNS) tumors among adults. Most adenomas are benign and exert their effect via excess hormone secretion or mass effect. Clinical presentation of pituitary adenoma varies based on their size and hormone secreted. Here, we review some of the most common types of pituitary adenomas, their clinical presentation, and current diagnostic and therapeutic strategies.

## 1. Introduction

The pituitary gland is located at the base of the brain, coming off the inferior hypothalamus, and weighs no more than half a gram. The pituitary gland is often referred to as the “master gland” and is the most important endocrine gland in the body because it regulates vital hormone secretion [1]. These hormones are responsible for vital bodily functions, such as growth, blood pressure, reproduction, and metabolism [2]. Anatomically, the pituitary gland is divided into three lobes: anterior, intermediate, and posterior. The anterior lobe is composed of several endocrine cells, such as lactotropes, somatotropes, and corticotropes, which synthesize and secrete specific hormones. The synthesis and release of these hormones are controlled by regulatory hormones secreted by the hypothalamus. Pituitary adenomas are located in the anterior lobe. The posterior lobe is an extension of the hypothalamus and releases hormones directly synthesized by the hypothalamus into the bloodstream [3].

Pituitary adenomas (PA) are non-metastasizing neoplasms that arise in the pituitary gland. Previously, PAs were classified based on their sizes. Those smaller than 10 mm were known as microadenoma and the rest as macroadenoma [4]. In recent years, the proteomic studies of PAs have led to a better appreciation of the different hormonal status shown by these tumors. As a result, PAs are classified as either functional or non-functional, based on their hormone-secreting capabilities. Patients with functional PAs present themselves with endocrine-related clinical phenotypes. Such phenotypes are associated with different hyperpituitarism or hypopituitarism symptoms depending on the hormone secretion levels impacted by the PA. Patients with non-functional PAs do not secrete any detectable hormones but are clinically active and may exhibit symptoms associated with mass effect, such as vision loss and headaches [4,5,6]. About one third of PAs are pituitary “incidentalomas”, given asymptomatology. They are most often detected incidentally during unrelated imaging or postmortem examinations [6].

This review summarizes the molecular characteristics of PAs, their clinical symptoms, and current treatment options. In the process, we also highlight improvements in our understanding of therapeutic interventions in the form of surgical, radiotherapy, and small-molecule drugs for PAs in the last decade.

## 2. Epidemiology

Central nervous system (CNS) tumors account for 1.4% of all newly diagnosed tumors. Among adults, PAs are the third most common CNS tumors accounting for 15% of all CNS tumors, with a vast majority of those being benign PAs [7]. The overall community prevalence, globally, of PA is estimated to be between 68 to 115 per 100,000 [8,9,10,11,12]. Data from studies looking at community prevalence of PA is summarized in Table 1. Due to slow growth rates, PAs tend to present insidiously with non-specific symptoms, so an accurate estimation of the prevalence remains a challenge. A meta-analysis based on autopsy and radiographic studies estimated that PAs develop in approximately 16.7% of the population. A significant fraction of these incidences are believed to be incidentalomas, which lack clinical symptoms and remain undiagnosed until an autopsy examination [4,13].

Among the clinically diagnosed cases of PAs, prolactinoma is the most common subtype and accounts for 40% to 66% of the cases. Non-functional PA is the second most common subtype (14% to 43%), followed by growth hormone secreting (GH)-PA and adrenocorticotropic hormone secreting (ACTH)-PA [8,9,10,11,12]. PA is also more common among females than males, with an incidence rate of 3.11 per 100,000 in females and 2.71 per 100,000 among males in the United States [7].

## 3. Classification of Pituitary Adenomas, Their Characteristics, and Treatment Strategies

Classification of PA by size (microadenoma versus macroadenoma) continues to be frequently used today but has limited impact on clinical decision-making as size does not always accurately predict symptoms or need for potential treatments. Larger PAs tend to more frequently have mass effect and symptoms, but an exact size that consistently produces symptoms does not exist. Clinical decision-making should be driven by signs and symptoms and not purely based on size. Hormonal production and immunohistochemical staining can be used to further classify PAs based on the predominant hormone and pituitary-specific biomarkers expressed by the tumor: prolactinoma, GH-secreting PAs, ACTH-secreting PAs, nonfunctional PAs, etc. For example, nonfunctional PAs can be categorized into eight subtypes: silent gonadotroph, corticotroph, somatotroph, thyrotroph, lactotroph, plurihormonal Pit-1, null-cell, and double/triple NFPAs based on expression of pituitary hormones and pituitary-specific transcription factors. However, further studies are needed to establish a strong correlation between histopathological factors and clinical behavior for PAs [14]. We hope the advancement of population-based genetic studies specific to PA tumors can expand our understanding into the genetics behind PAs and lead to improved classification and clinical outcome predictions.

## 4. Prolactin-Secreting Pituitary Adenoma (Prolactinomas)

Prolactinomas are PAs associated with the benign neoplastic proliferation of lactotroph cells in the anterior pituitary, leading to elevated prolactin secretion [15]. They are the most prevalent pituitary neoplasia, accounting for 40% to 66% of all PAs, with higher prevalence in females [16].

### 4.1. Clinical Presentation

Prolactin has a wide range of physiological effects. Its two primary functions include milk production and development of mammary glands [15]. Prolactin secretion by lactotrophs is stimulated by estrogen, thyrotropin-releasing hormones (TRH), and serotonin and it is restricted mostly by the tonic secretion of dopamine from the hypothalamus [17]. Among prolactinoma patients, clinical presentations are associated with either excessive autonomic production of prolactin by the lactotroph cells, leading to hyperprolactinemia, or mass effect caused by tumor size [18]. Mass effects are often observed with macroprolactinomas and manifest themselves as headaches due to dural irritation, visual disturbances due to the compression of the optic chiasm, and hypopituitarism due to compression of the pituitary stalk [5,19].

In hyperprolactinemia, excess prolactin leads to inhibition of the gonadotropin-releasing hormone (GnRH) from the hypothalamus. The exact mechanism of GnRH inhibition by prolactin in humans is still poorly understood, with some mouse models suggesting an alteration in intracellular signaling within GnRH neurons by prolactin [20]. The inhibition of GnRH by excess prolactin disrupts the hypothalamic-pituitary-gonadal axis (HPG). GnRH inhibition decreases downstream secretion of the follicle-stimulating hormone (FSH) and luteinizing hormone (LH) by the anterior pituitary, resulting in gonadal and sexual dysfunction [15,21]. As a result, men with hyperprolactinemia often present with infertility, impotence, erectile dysfunction, and decreased libido [19,22,23]. On the other hand, FSH and LH absence disrupts menstruation, leading to amenorrhea in premenopausal women [19]. Hypogonadism, as a result of hyperprolactinemia, also decreases bone density in both males and females [24].

Elevated prolactin levels may also be seen secondary to renal disease, pregnancy, primary hypothyroidism, and drug-induced hyperprolactinemia [25,26]. Non-prolactin secreting PAs can also cause mild hyperprolactinemia via the pituitary stalk effect. Large non-prolactin secreting PAs can compress the portal vessels or damage the pituitary stalk, disrupting the hypothalamic control over pituitary hormone secretion, leading to hyperprolactinemia and pituitary insufficiency [27]. Hence, the diagnosis of prolactinomas requires a detailed history, physical exam, pregnancy test, TSH test, and liver and renal function test to rule out non-tumorigenic causes of hyperprolactinemia [28].

### 4.2. Diagnosis

**Figure 1:**
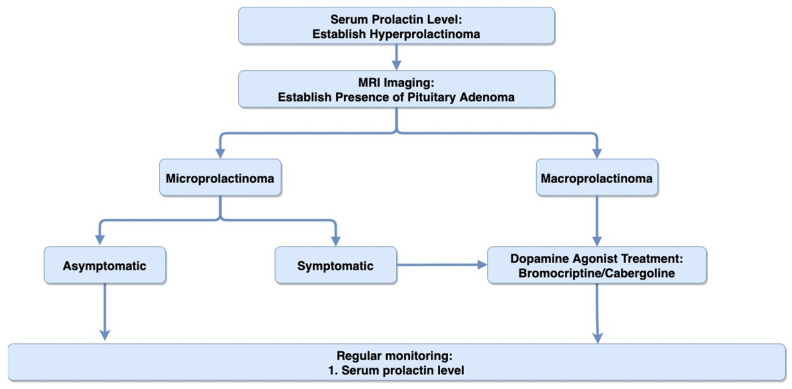
Diagnosis and Treatment Algorithm: Diagram representing the suggested algorithm for the diagnosis and treatment of prolactinoma.

Diagnosis of prolactinoma involves measurement of serum prolactin followed by MRI imaging to confirm the presence of pituitary growth. Prolactin levels below 25 µg/L and 20 µg/L in females and males, respectively, are considered to be normal [29]. Prolactin levels often correlate with tumor size, so those greater than 500 µg/L likely indicate the presence of a prolactin-secreting macroadenoma. For these very high levels, most laboratories must perform “dilution” studies to ascertain the exact degree of hyperprolactinemia. Levels greater than 250 µg/L may suggest the presence of microprolactinoma. However, high serum levels of prolactin can also be seen with pregnancy (range: 200–500 µg/L), medication-induced hyperprolactinemia (range: 25–200 µg/L), or the “stalk effect” (range: 25–250 µg/L), where the inhibitory dopamine secretion is blocked and causes a rise in prolactin [29,30,31]. Hence, confirmatory prolactinoma diagnosis requires both biomarker measurements, indicating sustained elevation of prolactin and radiographic imaging (MRI), showing pituitary adenoma [19]. Figure 1 summarizes the suggested algorithm for diagnosis and treatment of prolactinoma.

### 4.3. Treatment

Treatment goals for prolactinoma include: (i) suppression of excess prolactin secretion and reversal of its clinical consequences (such as infertility, decreased libido, and bone mass density); (ii) reduction of tumor size and relief of mass effect (such as vision deficit and headaches); (iii) preservation of remaining pituitary function; and (iv) prevention of disease progression and recurrence [17,32]. While the overall treatment goals for microprolactinomas and macroprolactinomas are the same, the reduction of tumor size takes priority over treating endocrine dysfunction in macroprolactinomas. Macroprolactinomas present with increased risk for neurological deficit (vision loss or headaches) and tumor invasion into adjacent cavernous sinuses. Hence, control of the tumor size is considered the primary treatment goal in macroprolactinomas, followed by hormonal control [32]. Regular monitoring of the serum prolactin level every 6–12 months is recommended over immediate treatment initiation in asymptomatic patients with microprolactinomas [33].

Fortunately, prolactinomas can be completely treated medically. Indeed, these are the only intracranial tumors that can be cured medically. Dopamine agonists are the first-line treatment for prolactinoma. Dopamine agonists bind to D2 receptors found on the surfaces of normal and tumorous lactotrophs. This binding alters the downstream signaling in lactotrophs and decreases prolactin secretion [17,32]. Currently, dopamine agonists bromocriptine and cabergoline are the two mainstay treatments for prolactinoma [34]. They both act as D2 receptor agonists and thus have similar side effects, such as headaches, dizziness, nausea, and vomiting [17]. Cabergoline has a longer half-life and requires fewer doses (0.5–1.5 mg/week) than bromocriptine (2–15 mg/day) [17,35,36,37]. Higher doses (up to 3.5 mg/week of cabergoline and 30 mg/day of bromocriptine) can be used for treatment in patients with resistant prolactinomas [17,35,36].

In a randomized and double-blinded study involving 459 women with hyperprolactinemia, 83% of those treated with cabergoline achieved normoprolactinemia compared to 59% for bromocriptine. Furthermore, there were fewer adverse effects and higher reproductive function recovery among subjects treated with cabergoline [38]. Similarly, male patients with hyperprolactinemia who took cabergoline showed a rapid decline in prolactin, earlier resolution of gonadal dysfunction, and improved libido compared to those who took bromocriptine [39]. Cabergoline has also been shown to be effective against bromocriptine resistant/intolerant prolactinomas [40,41,42].

These studies suggest that cabergoline is more efficacious than bromocriptine, but the exact mechanism of action is not well-understood. A potential explanation can be attributed to the fact that, compared to bromocriptine, cabergoline has (i) higher affinity for dopamine receptor binding site, (ii) longer drug-receptor interaction, and (iii) delayed elimination from the pituitary [31,43]. Thus, cabergoline is widely accepted as the preferred treatment choice for prolactinoma. However, bromocriptine is cheaper and hence can be preferred in resource-limited settings [17,19,31,44,45,46].

Dopamine agonist therapy should be started at the lowest dose and adjusted over time based on patient response [31,46]. Treatment response can be monitored by measuring serum prolactin levels. Once serum prolactin levels have normalized, they can be monitored every 3–6 months in the first year and every 6–12 months thereafter. In patients with visual field defects, regular vision examinations and MRIs are also recommended [19,31]. MRIs can be discontinued once maximal size reduction has been documented, after which patients are monitored with prolactin levels. Visual field testing can be discontinued once vision is normalized or remains stable [47].

Cardiac valve fibrosis is a rare side-effect seen among cabergoline-treated Parkinson’s patients [48,49]. The cumulative dose of cabergoline administered in Parkinson’s patients (range of 2600–6700 mg) is significantly higher than the cumulative dose given to treat prolactinoma (range of 200–500 mg) [50]. This lowered cumulative dose could potentially be the reason why studies have shown no relationship between cabergoline-treated prolactinoma patients and increased incidence of valvular dysfunction [50,51,52,53]. However, as a precaution, current guidelines recommend a close surveillance for valvular defect through regular echocardiogram at 6–12 month intervals [54]. Further research is needed to establish the clinical efficacy and cost-effectiveness of this guideline.

Until recently, dopamine agonist therapy was considered to be a life-long intervention [55]. However, recent studies have shown the possibility of withdrawing dopamine agonist while maintaining remission. In a prospective study with 200 hyperprolactinemia patients (52% microprolactinoma and 35% macroprolactinoma), cabergoline was phased out among patients who met the following criteria: (i) normalization of serum prolactin; (ii) absent tumor or reduction in tumor volume by at least 50% upon imaging; and (iii) no invasion of cavernous sinus and tumor located at least 5 mm from the optic chiasm. Within 2–5 years of withdrawal of dopamine agonist, hyperprolactinemia recurred among 31% of patients with microprolactinoma and 36% of patients with macroprolactinoma [56]. Another retrospective study evaluated recurrence among 89 patients with microprolactinoma that had abruptly withdrawn from dopamine agonist following 3 years of treatment on average. Among these patients, recurrence of prolactinoma was seen in 64% of patients and occurred on average 9.6 months after drug withdrawal [57]. Based on these studies, the current guidelines for the treatment of prolactinoma recommend a gradual withdrawal of dopamine agonist treatment in patients with at least 2 years of normoprolactinoma and reduction in tumor size greater than 50%. Following withdrawal, the guidelines recommend close monitoring of patients with serum prolactin measurements and MRI [19].

## 5. Acromegaly (Growth Hormone-Secreting Pituitary Adenoma)

GH-secreting PA is associated with neoplastic proliferation of somatotroph cells, which leads to growth hormone hypersecretion and, eventually, acromegaly [58]. Approximately 95% of acromegaly cases are caused by monoclonal GH-secreting pituitary adenoma, and the rest are caused by either hypothalamic or neuroendocrine tumors [59].

### 5.1. Clinical Presentation

The clinical symptoms of GH-secreting PA include mass effect due to local enlargement and compression from the adenoma and systemic complications due to over-secretion of the growth hormone [60]. Growth hormone receptors are predominately found on the liver and cartilage [61]. Growth hormones bind to these receptors and stimulate the liver to secrete insulin-like growth factor 1 (IGF-1), thereby elevating systemic and local levels of IGF-1 [62]. Hence, the clinical symptoms seen with GH-secreting PA result from both the direct elevation of GH and indirect elevation of IGF-1 [60].

At the time of diagnosis, patients typically present with excessive growth of hands and feet and coarsened facial features. Other acromegaly-associated clinical markers include changes to skin (hyperhidrosis and increased skin tags), osteoarthritis, carpal tunnel syndrome, colon polyps, reproductive disorders (menstrual disturbances or erectile dysfunction), respiratory failure, vision changes, and cardiovascular disease (hypertension, arrhythmia, and acromegalic cardiomyopathy, leading to congestive heart failure) [60,63,64].

### 5.2. Diagnosis

Diagnosis of GH-secreting PA involves measurements of IGF-1, followed by oral glucose tolerance testing (OGTT) for GH suppression and MRI imaging [59]. IGF-1 measurement is recommended for patients presenting with typical clinical manifestation of acromegaly (acral and facial features) or patients without typical manifestation of acromegaly but presenting with several associated conditions, such as diabetes mellitus, arthritis, hyperhidrosis, carpel tunnel syndrome, and hypertension. In patients with elevated serum IGF-1, OGTT is performed. In healthy patients, OGTT suppresses GH secretion. Hence, a lack of suppression of GH to <1 µg/L is used to confirm the biochemical diagnosis of acromegaly. Once a biochemical diagnosis of acromegaly has been established, MRI imaging is used to determine the size and location of the tumor. Vision tests are also recommended if adenomas are present near the optic chiasm [59,65,66].

Patients with acromegaly have an increased risk for colonic neoplasia (pooled risk ratio of 2.04), so a colonoscopy is recommended at the time of diagnosis [59,67]. Similarly, patients with acromegaly have a higher risk of thyroid cancer (risk ratio of 7.2) and thyroid nodular disease (risk ratio of 2.1), so acromegaly patients with palpable thyroid nodularity must be screened for thyroid cancer using thyroid ultrasound [59,68].

Clinical symptoms associated with acromegaly develop insidiously, causing significant diagnostic delay [69]. A retrospective study with 324 patients who had acromegaly between 1981–2006 showed that, while nearly 96.3% of patients had a history of facial and acral changes associated with acromegaly, these symptoms developed insidiously and were often overlooked by patients and their family members and physicians, and patients were only diagnosed once serious acromegaly-associated comorbidities had developed [70]. The current estimate suggests that the diagnosis of acromegaly is often delayed by 5 additional years since the onset of symptoms [69,70].

Delay in diagnosis leads to poor prognosis and higher healthcare burden. At the time of detection, approximately 80% of GH-producing PAs are macroadenomas, which further increases the risk for adverse surgical outcomes [59,60,69]. While it is widely accepted that we need new diagnostic strategies to reduce the gap between symptom initiation and diagnosis, studies that evaluated the benefits of screening using IGF-1, especially among patients with acromegaly associated comorbidities (such as sleep apnea, diabetes, or hypertension), deemed screening for acromegaly as cost-ineffective, possibly due to the low prevalence of acromegaly in the general population [71,72].

**Figure 2:**
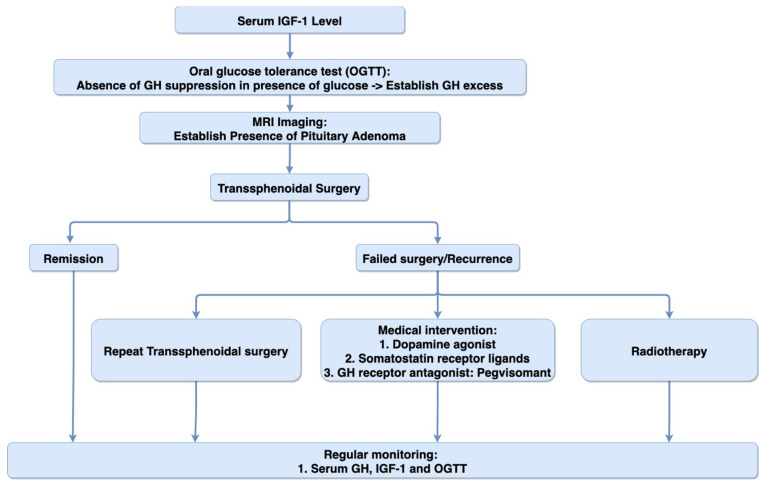
Diagnosis and treatment algorithm. Diagram representing the suggested algorithm for the diagnosis and treatment of GH-secreting pituitary adenoma.

However, in recent years, computer software has been developed that can detect acromegaly using patients’ photographs. In a study with 49 facial photographs (24 acromegaly patients, 25 normal subjects), the software was able to detect acromegaly with an accuracy of 86%, which was significantly higher than physicians’ accuracy of 26% [73]. Several machine-learning programs have also been developed to detect acromegaly based on patients’ photographs with high sensitivity and specificity [74]. These computer-aided methods are cost-effective and have the potential to be used for timely diagnosis of acromegaly in the future [72]. Figure 2 summarizes the suggested algorithm for diagnosis and treatment of GH-producing PA.

### 5.3. Treatment

Treatment goals for GH-secreting PA include: (i) normalization of GH and IGF-1 levels; (ii) reduction of tumor size; and (iii) reduction of acromegaly-associated comorbidities [66]. The current gold-standard method of treating GH-secreting PA is transsphenoidal surgery, except in patients with surgical contraindications. Treatment response after surgery is monitored with regular measurement of serum GH and IGF-1 levels and OGTT every 6–12 months [66,75,76]. Approximately 84% to 91% of patients with microadenoma and 46% to 64% of patients with macroadenoma show remission from acromegaly after transsphenoidal surgery [77,78,79]. Several studies have shown improved surgical outcomes in patients that received pre-operative somatostatin analog; however, further research is needed to understand the short-term and long-term benefits of this approach [80,81]. Currently, the treatment approach for patients with persistent disease, even after surgery, include: (i) repeat resection; (ii) medical intervention; and (iii) radiotherapy [59,66]. A retrospective study about the effectiveness of repeat resection analyzed surgical outcomes of 28 patients who underwent secondary resection for persistent or recurring GH-secreting PA and found that the overall remission rate after repeat resection was 57%, with non-invasive adenomas showing a remission rate close to 90% without any serious morbidities or mortality [82]. Other studies have since confirmed these results [83,84,85]. Repeat resection is also a cost-effective treatment for persistent acromegaly. On average, transsphenoidal surgery costs $39,311, which is cheaper than both radiotherapy, with an average cost of $56,356, and medical intervention, which can range anywhere between $1,578,567 and $2,620,833, depending on the medication used [83]. These studies highlight the efficiency and cost-effectiveness of repeat resection as a tool for long-term control of persistent acromegaly, especially for persistent non-invasive GH-secreting adenomas [86].

Medical therapy for GH-secreting adenomas is prescribed in cases of either surgical contraindication or persistent disease following surgery. Dopamine agonists, such as cabergoline, are the preferred treatment options in cases with modest elevation of IGF-1 and mild GH excess symptoms [59]. For patients with significant persistent acromegaly (i.e., severe GH excess symptoms), somatostatin receptor ligands (SRLs), or pegvisomant (GH receptor antagonist) are preferred. SRLs, such as octreotide and lanreotide, are often the first-line drug for medical intervention in patients with significant persistent acromegaly [59,61]. These drugs are able to achieve biochemical control in around 55% of the patients; however, meta-analysis shows a wide variability in response and efficacy of SRLs [87,88]. The most common side effects seen with SRLs include GI symptoms, such as abdominal cramps, flatulence, and diarrhea [59,61].

Radiotherapy is reserved as a third-line therapy following either a persistence recurrence after surgery or intolerance to medical therapy [61,66]. Radiotherapy can be delivered in two schedules, depending on the size and location of the tumor. Stereotactic radiosurgery involves delivery of a single high dose of radiation in a single treatment visit. However, if the tumor is adjacent to optic nerves or optic chiasm, a high dose of radiation can damage the surrounding brain structures. In such cases, fractioned radiation is preferred, where small doses of radiation are administered 5 days/week for 5–6 weeks, which lowers the likelihood of injury to surrounding tissues. While fractioned radiation reduces the risk of radiation-induced optic neuropathy, it is slower in relieving symptoms [89,90,91]. Overall, stereotactic radiosurgery is preferred over fractioned radiation because it has better remission rate and lower side effects [89,92,93].

## 6. Cushing’s Syndrome (ACTH-Secreting Pituitary Adenoma)

ACTH-secreting PAs are associated with an excessive secretion of adrenocorticotropic hormone (ACTH). Excess ACTH leads adrenal cortex to hypersecrete cortisol, leading to Cushing’s syndrome. ACTH-secreting PAs account for 70% of all endogenous Cushing’s syndrome [94,95].

Endogenous Cushing’s syndrome can be divided into two types: corticotropin-dependent and corticotropin-independent. Corticotropin-dependent causes of Cushing’s syndrome include ACTH-secreting PA, ectopic-ACTH secreting tumor, and corticotrophin-releasing tumor. Corticotropin-independent endogenous Cushing’s syndrome is due to adrenal overproduction of cortisol, seen secondary to adrenal tumor or bilateral adrenal hyperplasia [94,96]. Cushing’s can also be caused by chronic exposure to exogeneous glucocorticoids, leading to exogeneous Cushing’s syndrome [94,97].

### 6.1. Clinical Presentation

Clinical presentation of Cushing’s syndrome is broad and can vary significantly between patients [98]. Typical presentation of the disease consists of weight gain with central obesity, fat deposition in the posterior region of the neck, described as “buffalo hump” formation, facial skin thickening, purple striae located mainly in the lateral abdominal region, and easy body bruising [94,96,99]. Patients often present with other Cushing-associated comorbidities. These include glucose intolerance, reproductive dysfunction (decreased libido in men and menstrual disturbances in women), osteoporosis, hypertension, and spontaneous ecchymoses [95,99].

### 6.2. Diagnosis

Diagnosis of ACTH-secreting PA involves establishing hypercortisolism, followed by tests performed to identify the cause for endogenous Cushing’s syndrome, and MRI imaging to check for a pituitary mass [99]. The most common cause of Cushing’s syndrome is exogenous glucocorticoid use. Hence, before implementing any lab-based test, a detailed medication history must be evaluated to rule out iatrogenic Cushing’s syndrome [97,100].

There are three screening tests to confirm hypercortisolism: (i) 24-h urinary free cortisol; (ii) low-dose dexamethasone suppression test; and (iii) late-night salivary cortisol [97,100]. While the end-goal of these three tests is the same, i.e., to measure cortisol, late-night salivary has the highest sensitivity and specificity and greatest ease of use, making it the most widely used screening test [97,101,102]. The current guidelines recommend at least two urine or salivary cortisol measurements to validate results and improve test confidence [100].

**Figure 3:**
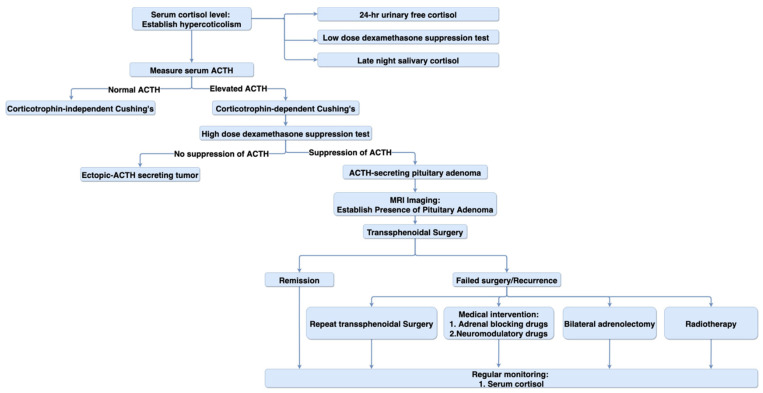
Diagnosis and treatment algorithm. Diagram representing the suggested algorithm for the diagnosis and treatment of ACTH-secreting pituitary adenoma.

After the initial differential diagnosis of hypercortisolism, secondary screening tests can be performed to identify the cause of endogenous Cushing’s syndrome [99,100,103]. A distinction between corticotrophin-dependent and corticotropin-independent Cushing’s syndrome can be made by the measurement of plasma ACTH levels [95]. High levels of ACTH indicate a corticotrophin-dependent process. A distinction between the two corticotrophin-dependent process (ACTH-stimulating PA and ectopic-ACTH secreting tumor) can be seen using the high-dose dexamethasone test. High-dose dexamethasone will suppress ACTH secretion from PA, but this negative-feedback is not seen with ectopic tumors. In patients with biochemical findings, indicating ACTH-secreting Cushing’s syndrome, MRI imaging can be done to provide a definitive diagnosis of ACTH-secreting PA [99]. These tumors are notoriously tiny and can be only a few millimeters in diameter, so may be difficult to see with a conventional MRI scan. MRI “sella” protocols can help identify tiny microadenomas. Using non-diagnostic MRI, most facilities recommend cavernous venous sinus sampling to identify the presence of a small ACTH-secreting PA. Figure 3 summarizes the suggested algorithm for diagnosis and treatment of ACTH-secreting PA.

### 6.3. Treatment

Transsphenoidal surgery is the first-line treatment for Cushing syndrome caused by ACTH-secreting PA. Treatment response after surgery is monitored with regular measurement of serum cortisol [104]. A literature review that considered 43 studies covering 6400 patients who received transsphenoidal surgery found the remission rate between 42% and 97% (with a median of 77.9%), with a higher remission rate among patents with microadenoma. The same review reported a recurrence rate between 0% and 47% (with a median of 11.5%) [105]. Similarly, other studies have found an average remission rate of 75% following transsphenoidal surgery [106,107,108]. For patients with incomplete resection, repeat resection is recommended as it led to remission in 65% of patients [100,109,110]. When transsphenoidal surgery is unsuccessful, medical therapy, radiotherapy, or bilateral adrenalectomy can be used for treatment [111].

Medical therapy can be divided into two categories: adrenal-blocking drugs (e.g., metyrapone, ketoconazole, and mitotane) and neuromodulatory drugs (e.g., valproate, cabergoline, and thiazolidinediones). Adrenal-blocking drugs act at the adrenal level and decrease the synthesis and secretion of cortisol from the adrenal glands. They exert their effect by inhibiting adrenal enzymes or adrenolytic activity (seen in mitotane). Neuromodulatory drugs act at the pituitary level to inhibit ACTH-secretion. However, none of these drugs have been shown to be effective in the long-term suppression of ACTH secretion in Cushing’s disease [99,112]. In recent years, pasireotide, which is a somatostatin analog, has been shown to significantly reduce cortisol levels in Cushing’s patients, but it may have limited application due to hyperglycemia-related adverse effects seen in approximately 72% of patients [113].

Total bilateral adrenalectomy can be used as a treatment in patients for whom repeated pituitary surgery is either contraindicated or has failed, and medical intervention alone cannot be used to control hypercortisolism [99]. Total bilateral adrenalectomy leads to rapid suppression of cortisol secretion, and, hence, leads to rapid symptom relief [114,115,116]. All adrenalectomized patients require life-long glucocorticoid and mineralocorticoid supplementation [97].

Radiotherapy is used as the last therapeutic intervention, often in a severe disease for which transsphenoidal surgery and medical intervention have failed to control Cushing’s syndrome [99]. Radiotherapy reduces tumors gradually and has a latency period between radiotherapy and biochemical control of around 12–24 months. Endocrine remission ranges from 46% to 100% in patients receiving fractionated radiotherapy and 10% to 100% in patients receiving stereotactic radiotherapy [117]. Although the majority of pituitary tumors are benign, some sub-groups may benefit from additional intervention in the form of small molecule chemotherapeutics or biologics, such as immunotherapy. A consensus guideline for various therapeutics options is provided by the European Society of Endocrinology [118].

## 7. Non-Functioning Pituitary Adenoma

Non-functioning pituitary adenomas are described as hormonally inactive adenomas. They can either be symptomatic, through mass effect that causes neurologic and pituitary dysfunction, or asymptomatic and are discovered incidentally, i.e., pituitary “incidentaloma”. A pituitary incidentaloma is defined as an unsuspected pituitary lesion discovered incidentally during imaging studies done for unrelated reasons [119].

### 7.1. Clinical Presentation

The symptoms in non-functioning PA arise due to mass effect, which in turn can be due to local pressure on non-pituitary tissue or pituitary compression, leading to hypothalamic and pituitary dysfunction [120]. Common symptoms include visual field defects due to the compression of optic chiasm and headaches due to irritation or increased stretch of the dura mater [121,122,123,124]. Hypopituitarism is a rare condition most often caused by PA. Compression of pituitary structures can lead to hypopituitarism, which present as hypogonadism and GH deficiency [123]. In some cases, cranial nerve dysfunction (CN III, IV, V1, V2, or VI) can occur due to lateral expansion of the tumor into the cavernous sinus [120,125].

### 7.2. Diagnosis

**Figure 4:**
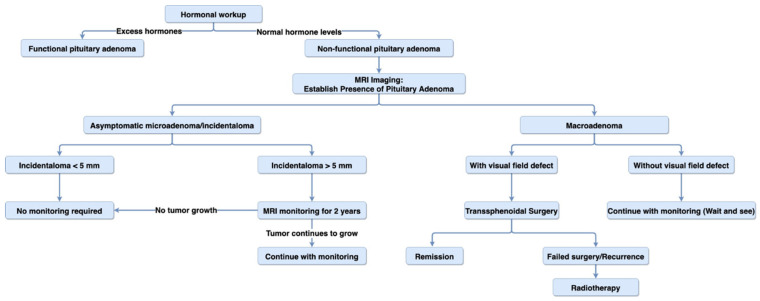
Diagnosis and treatment algorithm. Diagram representing the suggested algorithm for the diagnosis and treatment of non-functional pituitary adenoma.

Any patient with suspicion of PA, due to mass effect or because it was discovered incidentally, requires hormonal workup, which identifies pathological secretions and helps to distinguish between functional and non-functional PA [126]. MRI can be used to assess the morphology and location of the adenoma, and if a tumor is present near the optic chiasm, a visual field examination is recommended [119,126]. In patients with macroadenoma, hypopituitarism assessment (i.e., screening for gonadotroph, corticotrophin, and thyrotrophin hormones) is recommended and can help with pre- and post-operative planning [126]. Figure 4 summarizes the suggested algorithm for diagnosis and treatment of non-functional pituitary adenoma.

### 7.3. Treatment

Treatment options for non-functional PA include active surveillance, transsphenoidal surgical resection, and radiotherapy [124]. Transsphenoidal surgery remains the first-line therapeutic intervention for non-functioning PAs that cause mass effect symptoms. A total of 80% to 90% of patients that undergo surgery experience vision improvement [127]. In cases where macroadenomas are not pushing on the optic chiasm, a wait-and-see approach (close surveillance) can be implemented [126,128]. The treatment strategy for asymptomatic pituitary incidentaloma varies. While some studies suggest a “watch and wait” approach for all microadenomas, other classify a treatment strategy based on microadenoma size: incidentaloma <5 mm does not require hormonal or radiographic surveillance, while incidentaloma >5 mm should be monitored for the next 2–5 years for tumor progression using MRI every 6–12 months. If the tumor continues to grow, the surveillance should continue and, depending on the size of the tumor and the symptoms, surgery can be recommended [124,126].

Radiotherapy is not recommended as a primary treatment option. It is reserved as a postoperative adjuvant therapy, especially for patients with incomplete surgical resection [124]. Recurrence of non-functional PA was lower in patients who received a combination of surgery and postoperative radiation, compared to those who did not receive postoperative radiation [129]. Common side effects of post-radiotherapy include hypopituitarism, which requires hormone replacement therapy (17% to 56% of patients), vision deterioration (on average 1.6% of patients), and secondary brain tumor (1.9% to 2.7% of patients; 9.4 times higher relative risk than the general population) [130,131,132]. As a result, postoperative radiotherapy administered on a case-by-case basis is preferred over widespread use [128,133,134].

## 8. Rare Pituitary Disorders

Thyrotropin-secreting pituitary adenomas are rare PAs that account for less than 1% of all PAs [135,136]. They are diagnosed using MRI and thyroid-panels and present with symptoms of mass effect and/or hyperthyroidism. First-line treatment includes transsphenoidal surgery, followed by either medical therapy (somatostatin analogs or dopamine agonist) or radiotherapy in case of remission or surgical resistance [137].

Similar to PAs, disorders, such as craniopharyngioma, rathke cleft cyst, and sarcoidosis, can also occur in the intrasellar and suprasellar regions. While these disorders are rare, they can present with symptoms similar to Pas, such as headaches, visual disturbances, and hypopituitarism [138,139]. MRI features, such as tumor shape, characteristics, and enhancement patterns, can help differentiate PAs from other rare pituitary disorders [138,140].

## 9. Future Direction

In this section, we present three areas for future research. Transsphenoidal surgery still remains the first-line treatment for most cases of PAs. However, the proximity of PAs to vital endocrine and vascular structures can inhibit full resection of the tumor. In practice, it is often difficult to separate normal pituitary tissues from abnormal tumor tissues, which can lead to post-operative side-effects, such as hypopituitarism [141]. In recent studies, use of fluorescence agents, such as indocyanine green, have been found to be useful during transsphenoidal surgery. Fluorescent agents accumulate in tumor tissues and improve the definition of tumor margins and surrounding structures, helping surgeons separate them from normal pituitary tissues. Thus, these agents can improve resection rates and reduce post-operative side-effects [142,143]. However, we need more research on fluorescence-guided transsphenoidal surgery to better assess their safety, efficacy, and role in improving postoperative clinical outcomes.

In recent years, proton therapy has been explored as a potential treatment for PAs, especially as an alternative to radiotherapy, which is often the default option for surgery or medication-resistant PAs [144]. Studies have shown that proton therapy can be a safe and effective method for targeted treatment of PA, with low toxicity. It also reduces the risk of secondary radiation-induced tumors, such as brain tumors seen with radiotherapy [130,144,145]. However, we need further randomized control trials to better establish the benefits of proton therapy.

Most cases of PA occur sporadically with no known pathogenesis. In recent years, there have been many studies about the molecular mechanism of PAs. One of those studies exploring PA pathogenesis noted an increased expression of bromodomain-containing protein 4 (BRD4) in non-functioning and GH-PAs. BRD4 is an epigenetic regulator that causes transcriptional activation of oncogenes, such as *c-myc* and *bcl2*, which lead to proliferation of PA cells. Both in vitro and in vivo studies suggest that treatment with a BRD4 inhibitor leads to a significant inhibition in PA proliferation. These studies highlight BRD4 as a potential therapeutic target for nonfunctioning and GH-PA [146]. While studies have identified several genes involved in PA pathogenesis, further research is needed to gain a comprehensive understanding of the pathogenesis behind PAs to discover novel targeted PA therapeutics [147,148,149].

## 10. Conclusions

Pituitary adenomas account for approximately 15% of all CNS tumors. Recent data suggest that approximately 40% to 66% of all PAs are prolactinoma, 14% to 43% are non-functional pituitary adenoma, and the rest are either GH-secreting adenomas or ACTH-secreting adenomas. While mass effects are common for all four of these subtypes, other symptoms vary, depending on the predominant hormone secreted by the pituitary adenoma. Dopamine agonists are the first-line treatments for prolactinoma, and for the other subtypes, transsphenoidal surgery is the first-line therapeutic intervention followed by medical interventions. Radiotherapy is reserved for surgery and medication-resistant pituitary adenomas. In patients with remission, regular surveillance with serum hormone levels, MRI scans, and visual field testing are recommended to timely detect tumor recurrence.

## Figures and Tables

**Table 1:** Summary of population-based studies showing the prevalence of pituitary adenoma.

Study	Demographic	Collection Period	Total PA Cases Identified	Prevalence (per 100,000)	Proportion of all PA (%)				Proportion of All PA (%)	
					PRL-PA ^3^	ACTH-PA ^4^	GH-PA ^5^	NF-PA ^6^	Macroadenoma	Microadenoma
Agustsson et al., ^1,2^ [8]	Iceland	58 years (1955–2012)	471 (190 M, 281 F)	116	39.9	11.3	5.7	43.1	54.8	41.2
Gruppetta et al., [9]	Malta	11 years (2000–2011)	316 (96 M, 220 F)	76	46.2	2.2	16.5	34.2	43.4	56.6
Daly et al., [10]	Belgium	Data as of 30 September 2005	68 (22 M, 46 F)	94	66.2	5.9	13.2	14.7	42.6	57.3
Fernandez et al., [11]	United Kingdom	Data as of 31 July 2006	63 (21 M, 42 F)	78	57	2	11	28	41.3	58.7
Raappana et al., [12]	Finland	17 years (1992–2007)	164 (47 M, 117 F)	68	51	3	8.5	37	54	46

^1^ Agustsson et al. found that it was not possible to confirm adenoma size in 19 cases. The sum of macro–and microadenomas is therefore not 100%. ^2^ Patients with negative imaging results were classified as having a microadenoma. ^3^ PRL = prolactin ^4^ ACTH = adrenocorticotropic hormone ^5^ GH = growth hormone ^6^ NF = nonfunctional.

## Data Availability

Not applicable.
